# Eliminating Schistosomes through Vaccination: What are the Best Immune Weapons?

**DOI:** 10.3389/fimmu.2015.00095

**Published:** 2015-03-09

**Authors:** Cristina Toscano Fonseca, Sergio Costa Oliveira, Clarice Carvalho Alves

**Affiliations:** ^1^Laboratório de Esquistossomose do Centro de Pesquisas René Rachou, Fundação Oswaldo Cruz, Belo Horizonte, Brazil; ^2^Instituto Nacional de Ciência e Tecnologia em Doenças Tropicais (INCT-DT), Conselho Nacional de Desenvolvimento Científico e Tecnológico, Ministério de Ciência Tecnologia e Inovação, Universidade Federal de Minas Gerais, Belo Horizonte, Brazil; ^3^Departamento de Bioquímica e Imunologia do Instituto de Ciências Biológicas, Universidade Federal de Minas Gerais, Belo Horizonte, Brazil

**Keywords:** schistosome vaccine, protective immunity, schistosomiasis, effector mechanisms, immune response

## Abstract

The successful development of vaccines depends on the knowledge of the immunological mechanisms associated with the elimination of the pathogen. In the case of schistosomes, its complex life cycle and the mechanisms developed to evade host immune system, turns the development of a vaccine against the disease into a very difficult task. Identifying the immunological effector mechanisms involved in parasite attrition and the major targets for its response is a key step to formulate an effective vaccine. Recent studies have described some promising antigens to compose a subunit vaccine and have pointed to some immune factors that play a role in parasite elimination. Here, we review the immune components and effector mechanisms associated with the protective immunity induced by those vaccine candidates and the lessons we have learned from the studies of the acquired resistance to infection in humans. We will also discuss the immune factors that correlate with protection and therefore could help to evaluate those vaccine formulations in clinical trials.

## Introduction

Vaccination is a great strategy to control and eradicate diseases (1) and there is no doubt that the development of a vaccine against schistosomiasis would have a massive impact on disease control and would be useful as a complementary tool to the disease eradication (2). Schistosomes in contrast to viruses and bacteria are complex parasites that pass through different life stages in different anatomic sites of its definitive host (3). The parasite has evolved to live for decades in the host, developing interesting strategies to evade host immune system [reviewed in Ref. (4)]. So developing an effective vaccine against schistosomes is a difficult task. But since the parasite does not replicate in its definitive host, even a partial reduction in parasite burden is believed to have an impact on disease control and eradication.

For decades, scientists have tried to develop an effective vaccine formulation against schistosomes, and although two candidates are under clinical trials, the search for new candidates and vaccine formulations are far from ending. During all those years of studies on schistosome vaccine development, some immunological mechanisms involved in parasite elimination have been proposed. Complement activation has been suggested to be involved in parasite elimination, but from what we know so far, only recently transformed schistosomes are killed by complement and 24 h schistosomula became refractory to death induced by complement activation (5). Since most of these studies on complement activation and parasite death were performed *in vitro*, the significance of complement activation to parasite death *in vivo* can be questioned. Schistosomes only get to host vessels 72 h after penetrating the host and by this time the parasite is already resistant to death induced by complement (6). Indeed, many evasion mechanisms developed by the parasite have been described and give support to the idea that the activation of the membrane attack complex might not be the major mechanism involved in parasite elimination (7–10).

Antibody-dependent cellular cytotoxicity (ADCC) is another immune mechanism that has been associated with parasite elimination. In individuals living in endemic areas for schistosomiasis, ADCC involving IgE, IgG, eosinophils, monocytes, and platelets was associated with the acquisition of resistance to reinfection (11–13). In mice, ADCC has been highlighted as the immune mechanism involved in parasite death in animals immunized with Smp-80 and GST (14–16). However, eosinophils may not be the major cell involved in ADCC in mice, since deficiency in this cell did not result in any changes in worm and egg burden after infection, demonstrating that eosinophils do not play major roles in parasite death (17).

Regardless of the mechanisms involved, antibodies are key players in the protective immunity induced by vaccines. Immunization of mice deficient in B cells impaired the protective response induced in wild-type animals by vaccination with irradiated cercariae (18). Also, transference of sera from mice immunized with schistosomula tegument (Smteg) or Smp-80 to a naïve recipient induce partial protection against challenge infection (19, 20). Other evidence of the importance of antibodies in the protective immune response induced by vaccination comes from the studies of Hewiston and coworkers (21). They demonstrated that the protective immune response induced by attenuated cercariae was abrogated in CD154-deficient mice. CD40–CD154 interaction is involved in eliciting a humoral immune response dependent on T cells (22). The inoculation of IL-12 together with the vaccine in these deficient mice restored all the cellular immune parameters in mice lung but failed to restore protection and antibody production (21).

Cellular immune responses are also important in parasite elimination. Immunization of C57BL-6 mice deficient on IFN-γ, and TNFRI impair or abrogate protection induced by vaccine (18, 23, 24). The role of IFN-γ and TNF-α in parasite killing seems to be related to nitric oxide production by macrophage. Immunization of mice deficient in the TNFRI with irradiated cercariae abrogates protection and impairs nitric oxide synthase (iNOS) expression in lung macrophages (24). Nevertheless, immunization of mice deficient in iNOS result only in partial reduction on the protective immunity induced by irradiated cercariae, indicating that nitric oxide is not the major factor involved in parasite death (25).

In BALC-c mice, deficiency in IL-4R expression abrogates protection induced by irradiated cercariae that can be restored by wild-type serum transference (26). Recently, protective immunity-associated Th2 profile was observed in outbred mice immunized with glyceraldehyde 3-phosphate dehydrogenase (SG3PDH) and peroxiredoxin (TPX) (27). IL-10 and IL-17 production seems to correlate negatively with protection. Blocking IL-10 with neutralizing antibodies enables protection against challenge infection in mice previously infected with *Schistosoma mansoni* and treated with praziquantel (28). In *S. japonicum* infection, blocking IL-17 with neutralizing antibodies enhances antibody production and protection in infected mice (29).

Although CD8+ cells are classically related to immune responses against intracellular pathogens, its role in schistosome elimination has been recently described (30). Immunization of mice with the *S. japonicum* 22.6/26GST coupled to Sepharose 4B bead induced a significant reduction in parasite burden that was associated with an increase in the number of activated CD8+ cells (30). These activated CD8+ cells were able to promote death of parasite carrying host MHCI molecules in its surface (30).

Coulson and Wilson (31) suggested that the major mechanism involved in parasite elimination after immunization with the irradiated cercariae vaccine was in fact the generation of an inflammatory focus in the lung of immunized mice that impairs parasite migration and therefore its transformation into adult worms (31). Evidence that support this hypothesis is given by histological examination of mouse’s lungs which demonstrates that the parasites in the inflammatory foci were alive and when recovered from the lung and transferred to a naïve recipient they developed into adult worms (31, 32).

Besides all the knowledge generated and described so far, the majority of the studies on immune mechanisms involved in schistosome elimination have been performed using the irradiated cercariae strategy that for security reason are not used in human trials. Currently, researchers are developing vaccine formulations based on one or a cocktail of parasite antigens and although there are many studies on different vaccine strategies, little is known about the effective protective mechanisms. In this review, the immune components and mechanisms elicited by different vaccine strategies using subunit formulations containing promising parasite antigens will be described. We will evaluate whether there is (are) immune factor(s) that correlates with protection. And this information might be used to rationally design a vaccine formulation and to suggest a strategy that better elicits these protective responses. These correlates of protection can also help to evaluate whether those vaccines are effective during clinical trials.

## The Immune Response Elicited by Promising Schistosome Antigens

Some of the schistosome antigens tested in pre-clinical trials emerged as promising candidates to compose an anti-schistosomiasis vaccine due to their ability to consistently induce protective immune responses in different animal models, under different formulations and vaccine strategies.

Two schistosome antigens are under clinical trials, the fatty acid-binding protein of 14 kDa from *S. mansoni*, Sm14 (33), and the glutathione-*S*-transferase of 28 kDa from *S. haematobium* (34). The *S. mansoni* tetraspanin 2, TSP-2, is now been produced under good manufacture practices (GMP) to soon be evaluated in Phase I clinical Trial (35).

### Glutathione *S*-transferase

The 28 kDa glutathione *S*-transferase of *S. mansoni*, Sm28GST, is one of the most promising vaccine candidates. Recognized as the enzyme glutathione *S*-transferase (36), Sm28GST was identified in tegument, parenchyma, and genital organs of schistosomula and adult worm (16, 37). In the vaccine protocol, Sm28GST purified protein was able to significantly reduce the worm burden in rats and mice (16). *In vitro* experiments suggested that this protection was related to the cytotoxic response since in the presence of anti-Sm28GST antibodies and mouse eosinophils, schistosomula can be killed through ADCC (15, 16). The importance of antibodies in worm elimination was evaluated by the passive transfer of specific antibodies which were able to induce protection against challenge infection (16). A study performed by Boulanger and colleagues (38) reinforced the immunoprotective potential of Sm28GST protein, by demonstrating that immunization of baboons with the recombinant protein together with aluminum hydroxide (Alum) confers up to 80% of protection.

Several studies demonstrated the importance of antibodies in parasite elimination in animals immunized with Sm28GST. Mouse immunization with one dose of rSm28GST plus bacillus Calmette-Guérin (BCG) or Alum, as adjuvants, conferred protection against *S. mansoni* infection, and induced significant production of specific IgG, IgA, and IgE (39). In another study, mice immunization with Sm28GST produced in recombinant *Mycobacterium bovis* BCG, regardless of the immunization route induced a vigorous production of IgG1, IgG2a, and IgG2b levels, which was associated with the neutralization of the Sm28GST enzymatic activity (40). Intradermal immunization with a DNA encoding Sm28GST, also induced a significant production of anti-Sm28GST IgG antibodies, mainly IgG2a and IgG2b, with an ability to kill schistosomula through ADCC mechanism (41). In addition to the antibodies, the cellular immunity is also critical to *Schistosoma* elimination. Mice immunized with the recombinant Sm28GST protein or with peptide derived from C-terminal region showed reduction in worm burden, in fibrosis, and in the number of eggs in the liver that were associated with high levels of IFN-γ (42, 43). In a DNA vaccine strategy, immunization of mice with *Sm28GST* co-delivered with an IL-18-encoding plasmid, also induced a strong IFN-γ production and result in a significant reduction in egg and worm burden, reinforcing the importance of the Th1 response to the protection induced by Sm28GST (44).

One important feature of Sm28GST is the existence of cross-reactivity with other *Schistosoma* species, including *S. haematobium*, *S. japonicum*, and *S. bovis* (45). This property can be explored in the context of vaccine development which can act to eliminate different *Schistosoma* species at the same time. In this sense, it was demonstrated that immunization of primates with rSm28GST protect against heterologous infection with *S. haematobium* (46). The Sh28GST (GST protein derived from *S. haematobium*) has the ability to confer protection in monkeys that showed a reduction in worm fecundity (47). The results of the studies with schistosome GST as antigen in vaccine formulations clearly demonstrate the importance of antibodies for anti-fecundity effect and parasite elimination, through neutralization and ADCC mechanisms, respectively. It is important to note that this antigen can induce protection by reducing worm burden or female fecundity and thus this vaccine formulation is efficient to limit infection and pathology.

### Sm14

Sm14 is a *S. mansoni* fatty acid-binding protein that might be involved in lipid uptake from the host (48). Due to its predicted function, Sm14 represent an interesting target for vaccines against schistosomes. Schistosomes are unable to synthesize fatty acids and sterols through *de novo* pathway and therefore require host lipids to maintain its complex membrane system and physiological function (49, 50). Sm14 is expressed in the cercariae, schistosomula, adult worm, and eggs and located in the parasite tegument and gut, both tissue that represent the interface between parasite and host (51).

The ability of rSm14 to protect against schistosomiasis was first demonstrated by Tendler and coworkers (52). In their study, immunization of mice with rSm14 alone or formulated with Freund’s adjuvant (FA) induced protection levels ranging from 50 to 68% (52). In rabbits, rSm14 plus FA elicited 89% protection against challenge infection (52). Interestingly, Sm14 was also able to protect mice from *Fasciola hepatica* infection thus demonstrating its potential to be used in a vaccine formulation against both parasites (52).

In the case of Sm14, the protective immune response is dependent on IFN-γ and TNF-α production since in mice deficient in those cytokines, immunization with the recombinant form of Sm14 fails to induce protection (23). The importance of cellular components in the protective immune response elicited by Sm14 is also shown by the ability of epitopes from Sm14 to induce proliferative response in lymphocytes from resistant individuals and to induce protection in mice (53, 54). Immunization of mice with *Sm14* gene also induced significant protection associated with antibody production and increased production of IFN-γ by spleen cells and lung lavage cells (55). In a DNA vaccine strategy, the use of *IL-12* as adjuvant induced a significant production of IL-10 and nitric oxide and failed to induce antibody production and protection (55). The role of antibodies in the protective immunity induced by Sm14 cannot be ruled out. Although so far no direct role for antibodies in parasite elimination have been described, all the successful vaccine formulation containing Sm14 induce significant antibody production (23, 54, 55). Therefore, the role of antibodies in the protective immunity induced by Sm14 is a key question that has to be addressed if they are to be used as correlates of protection in future clinical trials.

### Tetraspanins

Tetraspanins are members of membrane-spanning proteins containing four transmembrane domains, three short intracellular domains, and two extracellular loops (EC1 and EC2) (56). In schistosomes, tetraspanins are located in the outer tegument, thus in contact with host immune system (57). The EC2 loop mediates protein–protein interactions (58) that are important to tetraspanin role in the molecular organization of cell membranes. Through interaction with many proteins, tetraspanins form a complex termed as tetraspanin-enriched microdomains (TEM) (59). The importance of tetraspanins to parasite survival was recently demonstrated using interference RNA technique (RNAi). Silencing *Tsp-1* and *Tsp-2* transcription resulted in significant reduction in the number of worm that reaches maturity in the mammalian host. Also, schistosomula treated with *Sm Tsp-2* double strand RNA displayed vacuolated and thinner tegument, demonstrating TSP-2 role in maintaining tegument integrity (60).

Immunization of mice with a recombinant form of TSP-1 and TSP-2 resulted in 29–38 or 53–61% reduction in worm burden, respectively. The protection was associated with an increased titer for IgG1 and IgG2a antibody (61). Immunization of mice with a vaccine formulation containing TSP-2, Alum, and CpG as adjuvant elicited a protection level ranging from 25 to 27%. In this vaccine formulation, although significant titers of IgG antibodies were observed, there was no clear association between antibody levels and parasite burden and also there was no significant increased production of cytokine specific for TSP-2 immunized group. In contrast, an increased level of IFN-γ, IL-4, and IL-10 were observed in mice immunized with a chimerical protein containing TSP-2 and the 5B region of the hookworm aspartic protease Na-APR-1 (62). Besides the great result observed against *S. mansoni* infection, TSP-2 orthologs in *S. japonicum* do not represent a good vaccine candidate, since in this species TSP-2 is extremely polymorphic (63).

### Smp-80

Another promising antigen is the large subunit of calpain, Smp-80. Schistosome calpain is composed of a smaller subunit of 28 kDa and a larger subunit of 78 kDa (64). The large subunit was described to be localized in the parasite surface (64). This subunit has proteolytic activity in the presence of Ca2+ (64) and plays an important role in immune evasion. Smp-80 is involved in the renewal/recycling of the parasite surface, an important evasion mechanism used by the parasite to escape from host immune system (65).

The large subunit of calpain has been evaluated in different vaccine formulations and strategies and the immune components produced in response to immunization have also been described. This antigen induced significant protection in mice, baboon, and hamster and protects against *S. mansoni*, *S. japonicum*, and *S. haematobium* (66–68). Immunization of mice with naked DNA containing *Smp-80* gene resulted in 39% of reduction in worm burden, the use of *IL-2* and *IL-12* gene as adjuvant increased the protection level to 57 and 45%, respectively. This increased protection was associated with an increased production of specific IgG2a and IgG2b, increased proliferative response, and IFN-γ production (69, 70). The use of *GM-CSF* and *IL-4* gene as adjuvant resulted in 42 and 44% reduction in worm burden associated with increased production of IL-4, IgG, IgG1, and IgG2b in *GM-CSF* immunized animals and IL-4 and IgG3 in *IL-4* immunized group (70, 71). When the recombinant form of Smp-80 is used as antigen either in its recombinant form or in a prime-boost regimen, higher titers of antibodies and significant production of IL-2 and IFN-γ are observed and this immune profile is associated with a reduction of 51 and 49% in worm burden (66). Therefore, the protective immune response induced by Smp-80 seems to correlate to a Th1 profile with increased IFN-γ and antibody production especially IgG2a.

Indeed in mice immunized with Smp-80, antibodies play an important role in the elimination of the parasite. Transference of sera from *Smp-80* immunized mice to a naïve recipient result in 31–45% reduction in worm burden after a challenge infection (20). Complement seems not to play a major role in the parasite elimination once immunization of mice deficient in C3 factor with Smp-80 did not result in significant reduction of the protection level observed in wild-type mice (72). ADCC instead seems to be the immune mechanism involved in parasite death, increased number of dead schistosomula was observed *in vitro* when these parasites were incubated with sera from Smp-80 plus CpG immunized mice in the presence of lung lavage cells or lung cells (14). The increased percentage of dead parasites was associated with production of nitric oxide suggesting that the production of this molecule might be involved in ADCC-induced parasite killing (14).

### Sm29

Sm29, other promising vaccine antigen, was identified by Cardoso and coworkers (73) using *in silico* analysis to identify in the *S. mansoni* transcriptome putative expressed proteins localized in the parasite tegument. The characterization of Sm29 demonstrated that this protein is expressed in the tegument of schistosomula and adult worm (74). The ability of Sm29 to induce protective immunity was assessed in mice with a vaccine formulation containing FA which resulted in a significant reduction of 51% in worm burden, 60% in intestinal eggs, and 50% in liver granuloma area, associated with a significant production of IgG, IgG1- and IgG2a-specific antibody, IFN-γ, TNF-α, and IL-10 cytokine production (74).

## Immune Response Induced by Multivalent Formulations

Schistosomes are complex parasites and thus the design of a multivalent vaccine against the parasite might enhance subunit vaccine efficacy. Recently, these multivalent vaccine formulations containing promising antigens have been tested. Sm29 was tested together with the SmTSP-2 in a multivalent chimeric recombinant vaccine in mice, aiming to enhance the single antigen vaccination efficacy. The vaccine formulation with a chimeric protein containing TSP-2 and the C-terminal part of Sm29 resulted in a small increase in the protection level induced by rSm29 alone from 20.36 to 34.83% (75), or by rTSP-2 alone from 27 to 34.83% (62, 75) when formulated with CpG-ODN plus alum. The chimeric protein induced a significant production of IgG and IgG2a-specific antibodies and a Th1 immune profile (75).

Another chimeric formulation combining Sm29 and Sm14 recombinant proteins was tested in vaccine protocol associated or not with poly (I:C)-adjuvant. Although immunization of Swiss mice with a subunit vaccine containing rSm14 or rSm29 alone did not induce significant reduction in adult worm, the vaccine formulation containing both rSm14 and rSm29 elicited 31 or 40% protection when it was formulated without adjuvant or with Poly (I:C), respectively (76).

The results observed in these multivalent formulations demonstrate that it is a promising strategy, but the choice of antigens that induce similar protective immune profile is a key step for the success of the vaccine formulation.

## What can we Learn from the Studies in Humans?

In endemic areas, the existence of naturally resistant individuals (77) that present persistently negative stool examination even if they are in constant contact with contaminated water enable the search for immune factors and biomarkers involved in resistance to *Schistosoma* infection.

Studies on human immune responses to Sm14, demonstrate that CD4+ T cells from naturally resistant individuals mounted a Th1-type of immune response to rSm14, based on IFN-γ and TNF-α production (78). Moreover, T-cell proliferative responses to rSm14 from these individuals were totally abrogated after treatment with anti-IFN-γ antibodies. These individuals also produce significant levels of IgG1 and IgG3 antibodies against Sm14, subclasses associated with parasite killing (79). Significant production of IgG1 and IgG3 specific for Sm29 and TSP-2 were also observed in individuals naturally resistant to *S. mansoni* infection (61, 80).

Human antibody responses to the large subunit of schistosomal calpain have also been associated with resistance. In humans infected with *S. japonicum*-presenting light infections, a strong reactivity to calpain was observed whereas in individuals with stronger infection, low reactivity to calpain was observed (81). In a study in an endemic area for schistosomiasis in Egypt, IFN-γ production in response to Sm14 and IgE and IgA antibodies against Sm28GST correlated with resistance to infection (82).

Grzych and colleagues (83) reinforce the pivotal role of the antibodies in protection. They show that IgA specific to Sm28GST were present in the sera from infected individuals before and after treatment with praziquantel and that these immunoglobulins have a key role in neutralizing the GST enzyme activity which resulted in impaired female fecundity.

Since 1998, Sh28GST recombinant protein plus aluminum hydroxide adjuvant has been tested in the human population. Partial results of the phase I clinical trials were published by Riveau and colleagues (34), in their study, no relevant side effects or toxicity following vaccine administration was observed. Humoral immune responses generated by the vaccine were characterized by high levels of IgG1 and IgG3 production and low levels of IgG2 and IgA. The cellular response profile was characterized by significant production of IL-5 and IL-13, resulting in a Th2 predominant response. The ability of antibodies to inhibit the enzymatic Sh28GST activity was also observed, corroborating with experimental studies (34).

## Conclusion and Future Perspective

Understanding the immunological mechanisms involved in parasite elimination during an infection is a key step toward the development of an effective vaccine. Here, we reviewed the immune components activated under different formulations containing antigens described as promising candidates to compose an anti-schistosomiasis vaccine (summarized in Table 1). Although for some of the antigens the immune mechanism involved in parasite death have been demonstrated, for others they are still to be identified. The biological role the target antigen plays in the survival of the parasite and the immunological components elicited by a protective formulation gives us an indication of what immune mechanisms might be involved in parasite death. Yet determining their involvement in protective immunity is still necessary. To address this question, the use of animals deficient in components of the immune system represents an interesting tool that should be further explored. Once those immune factors that correlate with protection are identified, they can be used as a biomarker of resistance in clinical trials.

**Table 1:** **Summary of the protection levels and immune components elicited by immunization**.

Antigen	Immunization strategy	Adjuvant	Protection level (%)	Humoral response	Cellular response	Reference
	DNA vaccine	None	39		Prolif. IFN-γ, IL-4	(67–69)
		IL-2	57	↑IgG, IgG2a and b	↑Prolif. ↑IFN-γ ↓IL-4	
		IL-12	45	↑IgG, IgG2a and b	↑Prolif. ↑IFN-γ ↓IL-4	
		IL-4	44	↑IgG3	Prolif. IFN-γ ↑IL-4	
		GM-CSF	42	↑IgG, IgG1	Prolif. IFN-γ ↑IL-4	
Smp-80	Prime E boost	Resiquimod (R848)	49	↑IgG, IgG1, IgG2a and b, IgG3, IgA, IgM	IL-2 and IFN-γ	(70)
	Recombinant protein	Resiquimod (R848)	51	↑IgG, IgG1, IgG2a and b, IgG3, IgA, IgM	IL-2 and IFN-γ	
		CpG-ODN	52.86	IgG, IgG1, IgG2b, IgG3, IgM	ADCC AND NO production	(14, 71)

	Recombinant protein	None	25			(22)
		FA	25	↑IgG2a	
Sm14		rIL-12	42.2	↑IgG2a	Protection is dependent on IFN-γ and TNF-α production
	DNA vaccine	None	40.5	IgG	IFN-γ by SC and BAL cells	(53)
	Synthetic peptides	FA + Padre	26–36.7	IgG1, IgG2a	IFN-γ IL-10	(52)

Sm29	Recombinant protein	FA	51	IgG1, IgG2a	IFN-γ, TNF-α, IL-10	(73)
		CpG-Alum	20	IgG, IgG1, IgG2a	IFN-γ	(74)

TSP-1	Recombinant protein	FA	29–38	IgG1, IgG2a	Not reported	(59)

TSP-2	Recombinant protein	FA	53–61	IgG1, IgG2a	Not reported	(59)
		Alum + CpG	25–27	IgG, IgG1^a^	IL-4, IFN-γ, and IL-10^b^	(60)

	Purified protein	FA	40–68.3	Not reported	Eosinophils (ADCC)	(16)
Sm28GST	Recombinant protein	Alum	46	Not significant	IL-2 and IFN-γ	(41)
	DNA vaccine	IL-18	23	Not significant	IFN-γ	(42)

Sh28GST	Recombinant protein	FA	77 (fecundity)	IgG and IgA	Not reported	(45)
		BCG	60	IgG	

*^a^No association with protection*;

*^b^production in response to infection and not to immunization; SC, spleen cells; BAL, broncho alveolar lavage; ↑ compared to vaccine formulation without adjuvant*.

## Conflict of Interest Statement

The authors declare that the research was conducted in the absence of any commercial or financial relationships that could be construed as a potential conflict of interest.

## References

[1] Andre FE Booy R Bock HL Clemens J Datta SK John TJ Vaccination greatly reduces disease, disability, death and inequity worldwide. Bull World Health Organ (2008) 86:140–6. 10.2471/BLT.07.040089 18297169 PMC2647387

[2] Oliveira SC Fonseca CT Cardoso FC Farias LP Leite LC. Recent advances in vaccine research against schistosomiasis in Brazil. Acta Trop (2008) 108:256–62. 10.1016/j.actatropica.2008.05.023 18577363

[3] Gryseels B. Schistosomiasis. Infect Dis Clin North Am (2012) 26:383–97. 10.1016/j.idc.2012.03.004 22632645

[4] Jenkins SJ Hewitson JP Jenkins GR Mountford AP. Modulation of the host’s immune response by schistosome larvae. Parasite Immunol (2005) 27:385–93. 10.1111/j.1365-3024.2005.00789.x 16179032 PMC1825761

[5] Novato-Silva E Nogueira-Machado JA Gazzinelli G. *Schistosoma mansoni*: comparison of the killing effect of granulocytes and complement with or without antibody on fresh and cultured schistosomula in vitro. Am J Trop Med Hyg (1980) 29:1263–7. 7446818 10.4269/ajtmh.1980.29.1263

[6] El Ridi R Tallima H. *Schistosoma mansoni* ex vivo lung-stage larvae excretory-secretory antigens as vaccine candidates against schistosomiasis. Vaccine (2009) 27:666–73. 10.1016/j.vaccine.2008.11.039 19056448

[7] Inal JM Sim RB. A *Schistosoma* protein, Sh-TOR, is a novel inhibitor of complement which binds human C2. FEBS Lett (2000) 470:131–4. 10.1016/S0014-5793(00)01304-1 10734221

[8] Pearce EJ Hall BF Sher A. Host-specific evasion of the alternative complement pathway by schistosomes correlates with the presence of a phospholipase C-sensitive surface molecule resembling human decay accelerating factor. J Immunol (1990) 144:2751–6. 1690776

[9] Ramalho-Pinto FJ. Decay accelerating factor (DAF) as the host antigen with protective activity to complement killing of schistosomula. Mem Inst Oswaldo Cruz (1987) 82:213–6. 10.1590/S0074-02761987000800037 2474124

[10] Marikovsky M Arnon R Fishelson Z. Proteases secreted by transforming schistosomula of *Schistosoma mansoni* promote resistance to killing by complement. J Immunol (1988) 141:273–8. 3132503

[11] Butterworth AE Sturrock RF Houba V Mahmoud AA Sher A Rees PH Eosinophils as mediators of antibody-dependent damage to schistosomula. Nature (1975) 256:727–9 10.1038/256727a0 1153011

[12] Joseph M Auriault C Capron M Ameisen JC Pancré V Torpier G IgE-dependent platelet cytotoxicity against helminths. Adv Exp Med Biol (1985) 184:23–33 10.1007/978-1-4684-8326-0_2 2994410

[13] Khalife J Dunne DW Richardson BA Mazza G Thorne KJ Capron A Functional role of human IgG subclasses in eosinophil-mediated killing of schistosomula of *Schistosoma mansoni*. J Immunol (1989) 142:4422–7. 2723436

[14] Torben W Ahmad G Zhang W Nash S Le L Karmakar S Role of antibody dependent cell mediated cytotoxicity (ADCC) in Sm-p80-mediated protection against *Schistosoma mansoni*. Vaccine (2012) 30:6753–8. 10.1016/j.vaccine.2012.09.026 23000221 PMC3488153

[15] Balloul JM Pierce RJ Grzych JM Capron A. In vitro synthesis of a 28 kilodalton antigen present on the surface of the schistosomulum of *Schistosoma mansoni*. Mol Biochem Parasitol (1985) 17:105–14. 10.1016/0166-6851(85)90131-8 2414656

[16] Balloul JM Grzych JM Pierce RJ Capron A. A purified 28,000 dalton protein from *Schistosoma mansoni* adult worms protects rats and mice against experimental schistosomiasis. J Immunol (1987) 138:3448–53. 3106483

[17] Swartz JM Dyer KD Cheever AW Ramalingam T Pesnicak L Domachowske JB *Schistosoma mansoni* infection in eosinophil lineage-ablated mice. Blood (2006) 108:2420–7. 10.1182/blood-2006-04-015933 16772607 PMC1895572

[18] Jankovic D Wynn TA Kullberg MC Hieny S Caspar P James S Optimal vaccination against *Schistosoma mansoni* requires the induction of both B cell- and IFN-γamma-dependent effector mechanisms. J Immunol (1999) 162:345–51. 9886405

[19] Melo TT Sena IC Araujo N Fonseca CT. Antibodies are involved in the protective immunity induced in mice by *Schistosoma mansoni* schistosomula tegument (Smteg) immunization. Parasite Immunol (2014) 36:107–11. 10.1111/pim.12091 24558655

[20] Torben W Ahmad G Zhang W Siddiqui AA. Role of antibodies in Sm-p80-mediated protection against *Schistosoma mansoni* challenge infection in murine and nonhuman primate models. Vaccine (2011) 29:2262–71. 10.1016/j.vaccine.2011.01.040 21277404 PMC3063403

[21] Hewitson JP Hamblin PA Mountford AP. In the absence of CD154, administration of interleukin-12 restores Th1 responses but not protective immunity to *Schistosoma mansoni*. Infect Immun (2007) 75:3539–47. 10.1128/IAI.00252-07 17485453 PMC1932915

[22] Grewal IS Flavell RA. CD40 and CD154 in cell-mediated immunity. Annu Rev Immunol (1998) 16:111–35. 10.1146/annurev.immunol.16.1.111 9597126

[23] Fonseca CT Brito CF Alves JB Oliveira SC. IL-12 enhances protective immunity in mice engendered by immunization with recombinant 14 kDa *Schistosoma mansoni* fatty acid-binding protein through an IFN-γamma and TNF-αlpha dependent pathway. Vaccine (2004) 22:503–10. 10.1016/j.vaccine.2003.07.010 14670333

[24] Street M Coulson PS Sadler C Warnock LJ McLaughlin D Bluethmann H TNF is essential for the cell-mediated protective immunity induced by the radiation-attenuated schistosome vaccine. J Immunol (1999) 163:4489–94. 10510391

[25] James SL Cheever AW Caspar P Wynn TA. Inducible nitric oxide synthase-deficient mice develop enhanced type 1 cytokine-associated cellular and humoral immune responses after vaccination with attenuated *Schistosoma mansoni* cercariae but display partially reduced resistance. Infect Immun (1998) 66:3510–8. 9673227 10.1128/iai.66.8.3510-3518.1998 PMC108380

[26] Mountford AP Hogg KG Coulson PS Brombacher F. Signaling via interleukin-4 receptor alpha chain is required for successful vaccination against schistosomiasis in BALB/c mice. Infect Immun (2001) 69:228–36. 10.1128/IAI.69.1.228-236.2001 11119510 PMC97876

[27] El Ridi R Tallima H. Vaccine-induced protection against murine schistosomiasis mansoni with larval excretory-secretory antigens and papain or type-2 cytokines. J Parasitol (2013) 99:194–202. 10.1645/GE-3186.1 22985345

[28] Wilson MS Cheever AW White SD Thompson RW Wynn TA. IL-10 blocks the development of resistance to re-infection with *Schistosoma mansoni*. PLoS Pathog (2011) 7:e1002171. 10.1371/journal.ppat.1002171 21829367 PMC3150278

[29] Wen X He L Chi Y Zhou S Hoellwarth J Zhang C Dynamics of Th17 cells and their role in *Schistosoma japonicum* infection in C57BL/6 mice. PLoS Negl Trop Dis (2011) 5:e1399. 10.1371/journal.pntd.0001399 22102924 PMC3216943

[30] Zhou Y Zhang H Sun XJ Zheng D Liang YJ Luo J Murine CD8(+)T cell cytotoxicity against schistosomula induced by inoculation of schistosomal 22.6/26GST coupled sepharose 4B beads. Vaccine (2012) 30:2440–7. 10.1016/j.vaccine.2012.01.068 22326902

[31] Coulson PS Wilson RA. Examination of the mechanisms of pulmonary phase resistance to *Schistosoma mansoni* in vaccinated mice. Am J Trop Med Hyg (1988) 38:529–39. 3152781 10.4269/ajtmh.1988.38.529

[32] Coulson PS The radiation-attenuated vaccine against schistosomes in animal models: paradigm for a human vaccine? Adv Parasitol (1997) 39:271–336 10.1016/S0065-308X(08)60048-2 9241818

[33] Oswaldo Cruz Foundation. Study to evaluate the safety of the vaccine prepared sm14 against schistosomiasis. ClinicalTrials.gov [Internet]. Bethesda (MD): National Library of Medicine (US) (2000). Available from: http://clinicaltrials.gov/ct2/show/study/NCT01154049

[34] Riveau G Deplanque D Remoué F Schacht AM Vodougnon H Capron M Safety and immunogenicity of rSh28GST antigen in humans: phase 1 randomized clinical study of a vaccine candidate against urinary schistosomiasis. PLoS Negl Trop Dis (2012) 6:e1704. 10.1371/journal.pntd.0001704 22802974 PMC3389022

[35] Curti E Kwityn C Zhan B Gillespie P Brelsford J Deumic V Expression at a 20L scale and purification of the extracellular domain of the *Schistosoma mansoni* TSP-2 recombinant protein: a vaccine candidate for human intestinal schistosomiasis. Hum Vaccin Immunother (2013) 9:2342–50. 10.4161/hv.25787 23899507 PMC3981843

[36] Taylor JB Vidal A Torpier G Meyer DJ Roitsch C Balloul JM The glutathione transferase activity and tissue distribution of a cloned Mr28K protective antigen of *Schistosoma mansoni*. EMBO J (1988) 7:465–72. 3284744 10.1002/j.1460-2075.1988.tb02834.x PMC454343

[37] Liu JL Fontaine J Capron A Grzych JM. Ultrastructural localization of Sm28 GST protective antigen in *Schistosoma mansoni* adult worms. Parasitology (1996) 113:377–91. 10.1017/S003118200006652X 8873477

[38] Boulanger D Reid GD Sturrock RF Wolowczuk I Balloul JM Grezel D Immunization of mice and baboons with the recombinant Sm28GST affects both worm viability and fecundity after experimental infection with *Schistosoma mansoni*. Parasite Immunol (1991) 13:473–90. 10.1111/j.1365-3024.1991.tb00545.x 1956696

[39] Grezel D Capron M Grzych JM Fontaine J Lecocq JP Capron A. Protective immunity induced in rat schistosomiasis by a single dose of the Sm28GST recombinant antigen: effector mechanisms involving IgE and IgA antibodies. Eur J Immunol (1993) 23:454–60. 10.1002/eji.1830230223 8436179

[40] Kremer L Riveau G Baulard A Capron A Locht C. Neutralizing antibody responses elicited in mice immunized with recombinant bacillus Calmette-Guérin producing the *Schistosoma mansoni* glutathione S-transferase. J Immunol (1996) 156:4309–17. 8666802

[41] Dupré L Poulain-Godefroy O Ban E Ivanoff N Mekranfar M Schacht AM Intradermal immunization of rats with plasmid DNA encoding *Schistosoma mansoni* 28 kDa glutathione S-transferase. Parasite Immunol (1997) 19:505–13. 10.1046/j.1365-3024.1997.d01-163.x 9427997

[42] Pancré V Wolowczuk I Bossus M Gras-Masse H Guerret S Delanoye A Evaluation of the effect of Sm28GST-derived peptides in murine hepatosplenic schistosomiasis: interest of the lipopeptidic form of the C-terminal peptide. Mol Immunol (1994) 31:1247–56. 10.1016/0161-5890(94)90075-2 7969186

[43] Pancre V Wolowczuk I Guerret S Copin MC Delanoye A Capron A Protective effect of rSm28GST-specific T cells in schistosomiasis: role of gamma interferon. Infect Immun (1994) 62:3723–30. 8063386 10.1128/iai.62.9.3723-3730.1994 PMC303023

[44] Dupré L Kremer L Wolowczuk I Riveau G Capron A Locht C. Immunostimulatory effect of IL-18-encoding plasmid in DNA vaccination against murine *Schistosoma mansoni* infection. Vaccine (2001) 19:1373–80. 10.1016/S0264-410X(00)00363-7 11163659

[45] Capron A Dessaint JP Capron M Ouma JH Butterworth AE. Immunity to schistosomes: progress toward vaccine. Science (1987) 238:1065–72. 10.1126/science.3317823 3317823

[46] Boulanger D Warter A Trottein F Mauny F Brémond P Audibert F Vaccination of patas monkeys experimentally infected with *Schistosoma haematobium* using a recombinant glutathione S-transferase cloned from *S. mansoni*. Parasite Immunol (1995) 17:361–9. 10.1111/j.1365-3024.1995.tb00903.x 8552409

[47] Boulanger D Warter A Sellin B Lindner V Pierce RJ Chippaux JP Vaccine potential of a recombinant glutathione S-transferase cloned from *Schistosoma haematobium* in primates experimentally infected with an homologous challenge. Vaccine (1999) 17:319–26. 10.1016/S0264-410X(98)00202-3 9987169

[48] Moser D Tendler M Griffiths G Klinkert MQ. A 14-kDa *Schistosoma mansoni* polypeptide is homologous to a gene family of fatty acid binding proteins. J Biol Chem (1991) 266:8447–54. 2022660

[49] Furlong ST. Unique roles for lipids in *Schistosoma mansoni*. Parasitol Today (1991) 7:59–62. 10.1016/0169-4758(91)90192-Q 15463424

[50] Meyer F Meyer H Bueding E Lipid metabolism in the parasitic and free-living flatworms, *Schistosoma mansoni* and *Dugesia dorotocephala*. Biochim Biophys Acta (1970) 210:257–66 10.1016/0005-2760(70)90170-0 4319989

[51] Brito CF Oliveira GC Oliveira SC Street M Riengrojpitak S Wilson RA Sm14 gene expression in different stages of the *Schistosoma mansoni* life cycle and immunolocalization of the Sm14 protein within the adult worm. Braz J Med Biol Res (2002) 35:377–81. 10.1590/S0100-879X2002000300014 11887217

[52] Tendler M Brito CA Vilar MM Serra-Freire N Diogo CM Almeida MS A *Schistosoma mansoni* fatty acid-binding protein, Sm14, is the potential basis of a dual-purpose anti-helminth vaccine. Proc Natl Acad Sci U S A (1996) 93:269–73. 10.1073/pnas.93.1.269 8552619 PMC40220

[53] Fonseca CT Cunha-Neto E Goldberg AC Kalil J de Jesus AR Carvalho EM Human T cell epitope mapping of the *Schistosoma mansoni* 14-kDa fatty acid-binding protein using cells from patients living in areas endemic for schistosomiasis. Microbes Infect (2005) 7:204–12. 10.1016/j.micinf.2004.10.012 15725385

[54] Garcia TC Fonseca CT Pacifico LG Durães Fdo V Marinho FA Penido ML Peptides containing T cell epitopes, derived from Sm14, but not from paramyosin, induce a Th1 type of immune response, reduction in liver pathology and partial protection against *Schistosoma mansoni* infection in mice. Acta Trop (2008) 106:162–7. 10.1016/j.actatropica.2008.03.003 18423420

[55] Fonseca CT Pacífico LG Barsante MM Rassi T Cassali GD Oliveira SC. Co-administration of plasmid expressing IL-12 with 14-kDa *Schistosoma mansoni* fatty acid-binding protein cDNA alters immune response profiles and fails to enhance protection induced by Sm14 DNA vaccine alone. Microbes Infect (2006) 8:2509–16. 10.1016/j.micinf.2006.06.008 16914349

[56] Maecker HT Todd SC Levy S. The tetraspanin superfamily: molecular facilitators. FASEB J (1997) 11:428–42. 9194523

[57] Braschi S Curwen RS Ashton PD Verjovski-Almeida S Wilson A. The tegument surface membranes of the human blood parasite *Schistosoma mansoni*: a proteomic analysis after differential extraction. Proteomics (2006) 6:1471–82. 10.1002/pmic.200500368 16447162

[58] Hemler ME. Tetraspanin functions and associated microdomains. Nat Rev Mol Cell Biol (2005) 6:801–11. 10.1038/nrm1736 16314869

[59] Hemler ME. Targeting of tetraspanin proteins-potential benefits and strategies. Nat Rev Drug Discov (2008) 7:747–58. 10.1038/nrd2659 18758472 PMC4737550

[60] Tran MH Freitas TC Cooper L Gaze S Gatton ML Jones MK Suppression of mRNAs encoding tegument tetraspanins from *Schistosoma mansoni* results in impaired tegument turnover. PLoS Pathog (2010) 6:e1000840. 10.1371/journal.ppat.1000840 20419145 PMC2855321

[61] Tran MH Pearson MS Bethony JM Smyth DJ Jones MK Duke M Tetraspanins on the surface of *Schistosoma mansoni* are protective antigens against schistosomiasis. Nat Med (2006) 12:835–40. 10.1038/nm1430 16783371

[62] Pearson MS Pickering DA McSorley HJ Bethony JM Tribolet L Dougall AM Enhanced protective efficacy of a chimeric form of the schistosomiasis vaccine antigen Sm-TSP-2. PLoS Negl Trop Dis (2012) 6:e1564. 10.1371/journal.pntd.0001564 22428079 PMC3302818

[63] Cai P Bu L Wang J Wang Z Zhong X Wang H. Molecular characterization of *Schistosoma japonicum* tegument protein tetraspanin-2: sequence variation and possible implications for immune evasion. Biochem Biophys Res Commun (2008) 372:197–202. 10.1016/j.bbrc.2008.05.042 18486598

[64] Siddiqui AA Zhou Y Podesta RB Karcz SR Tognon CE Strejan GH Characterization of Ca(2+)-dependent neutral protease (calpain) from human blood flukes, *Schistosoma mansoni*. Biochim Biophys Acta (1993) 1181:37–44. 10.1016/0925-4439(93)90087-H 8457603

[65] Silva EE Clarke MW Podesta RB. Characterization of a C3 receptor on the envelope of *Schistosoma mansoni*. J Immunol (1993) 151:7057–66. 8258710

[66] Ahmad G Zhang W Torben W Noor Z Siddiqui AA. Protective effects of Sm-p80 in the presence of resiquimod as an adjuvant against challenge infection with *Schistosoma mansoni* in mice. Int J Infect Dis (2010) 14:e781–7. 10.1016/j.ijid.2010.02.2266 20630783 PMC2941550

[67] Zhang R Yoshida A Kumagai T Kawaguchi H Maruyama H Suzuki T Vaccination with calpain induces a Th1-biased protective immune response against *Schistosoma japonicum*. Infect Immun (2001) 69:386–91. 10.1128/IAI.69.1.386-391.2001 11119528 PMC97894

[68] Siddiqui AA Siddiqui BA Ganley-Leal L. Schistosomiasis vaccines. Hum Vaccin (2011) 7:1192–7. 10.4161/hv.7.11.17017 22048120 PMC3323497

[69] Siddiqui AA Phillips T Charest H Podesta RB Quinlin ML Pinkston JR Enhancement of Sm-p80 (large subunit of calpain) induced protective immunity against *Schistosoma mansoni* through co-delivery of interleukin-2 and interleukin-12 in a DNA vaccine formulation. Vaccine (2003) 21:2882–9. 10.1016/S0264-410X(03)00159-2 12798631

[70] Siddiqui AA Pinkston JR Quinlin ML Kavikondala V Rewers-Felkins KA Phillips T Characterization of protective immunity induced against *Schistosoma mansoni* via DNA priming with the large subunit of calpain (Sm-p80) in the presence of genetic adjuvants. Parasite (2005) 12:3–8. 10.1051/parasite/2005121003 15828575

[71] Siddiqui AA Phillips T Charest H Podesta RB Quinlin ML Pinkston JR Induction of protective immunity against *Schistosoma mansoni* via DNA priming and boosting with the large subunit of calpain (Sm-p80): adjuvant effects of granulocyte-macrophage colony-stimulating factor and interleukin-4. Infect Immun (2003) 71:3844–51. 10.1128/IAI.71.7.3844-3851.2003 12819068 PMC161986

[72] Karmakar S Zhang W Ahmad G Alam MU Winn R Torben W Complement plays a minimal role in Sm-p80-mediated protection against *Schistosoma mansoni*. Hum Vaccin Immunother (2014) 10:640–7. 10.4161/hv.27576 24374377 PMC4130258

[73] Cardoso FC Pinho JM Azevedo V Oliveira SC. Identification of a new *Schistosoma mansoni* membrane-bound protein through bioinformatic analysis. Genet Mol Res (2006) 5:609–18. 17183472

[74] Cardoso FC Macedo GC Gava E Kitten GT Mati VL de Melo AL *Schistosoma mansoni* tegument protein Sm29 is able to induce a Th1-type of immune response and protection against parasite infection. PLoS Negl Trop Dis (2008) 2:e308. 10.1371/journal.pntd.0000308 18827884 PMC2553283

[75] Pinheiro CS Ribeiro AP Cardoso FC Martins VP Figueiredo BC Assis NR A multivalent chimeric vaccine composed of *Schistosoma mansoni* SmTSP-2 and Sm29 was able to induce protection against infection in mice. Parasite Immunol (2014) 36:303–12. 10.1111/pim.12118 24749785

[76] Ewaisha RE Bahey-El-Din M Mossallam SF Amer EI Aboushleib HM Khalil AM Combination of the two schistosomal antigens Sm14 and Sm29 elicits significant protection against experimental *Schistosoma mansoni* infection. Exp Parasitol (2014) 145:51–60 10.1016/j.exppara.2014.07.010 25092439

[77] Correa-Oliveira R Pearce EJ Oliveira GC Golgher DB Katz N Bahia LG The human immune response to defined immunogens of *Schistosoma mansoni*: elevated antibody levels to paramyosin in stool-negative individuals from two endemic areas in Brazil. Trans R Soc Trop Med Hyg (1989) 83:798–804. 10.1016/0035-9203(89)90334-9 2617649

[78] Brito CF Caldas IR Coura Filho P Correa-Oliveira R Oliveira SC CD4+ T cells of schistosomiasis naturally resistant individuals living in an endemic area produce interferon-gamma and tumour necrosis factor-alpha in response to the recombinant 14KDA *Schistosoma mansoni* fatty acid-binding protein. Scand J Immunol (2000) 51:595–601 10.1046/j.1365-3083.2000.00710.x 10849370

[79] Brito CF Fonseca CT Goes AM Azevedo V Simpson AJ Oliveira SC. Human IgG1 and IgG3 recognition of *Schistosoma mansoni* 14kDa fatty acid-binding recombinant protein. Parasite Immunol (2000) 22:41–8. 10744504

[80] Cardoso FC Pacífico RN Mortara RA Oliveira SC. Human antibody responses of patients living in endemic areas for schistosomiasis to the tegumental protein Sm29 identified through genomic studies. Clin Exp Immunol (2006) 144:382–91. 10.1111/j.1365-2249.2006.03081.x 16734606 PMC1941986

[81] Zhang R Suzuki T Takahashi S Yoshida A Kawaguchi H Maruyama H Cloning and molecular characterization of calpain, a calcium-activated neutral proteinase, from different strains of *Schistosoma japonicum*. Parasitol Int (2000) 48:232–42. 10.1016/S1383-5769(99)00024-0 11227763

[82] Al-Sherbiny M Osman A Barakat R El Morshedy H Bergquist R Olds R. In vitro cellular and humoral responses to *Schistosoma mansoni* vaccine candidate antigens. Acta Trop (2003) 88:117–30. 10.1016/S0001-706X(03)00195-5 14516923

[83] Grzych JM Grezel D Xu CB Neyrinck JL Capron M Ouma JH IgA antibodies to a protective antigen in human *Schistosomiasis mansoni*. J Immunol (1993) 150:527–35. 8419485

